# Management of *BRCA*-associated breast cancer patients in low and middle-income countries: a review

**DOI:** 10.3332/ecancer.2024.1744

**Published:** 2024-08-22

**Authors:** Fen Saj, Shona Nag, Nita Nair, Bhawna Sirohi

**Affiliations:** 1 Department of Medical Oncology, Balco Medical Centre-Vedanta Medical Research Foundation, Raipur 493661, India; 2 Department of Medical Oncology, Sahyadri Hospital, Pune 411004, India; 3 Department of Surgical Oncology, Apollo Hospitals, Mumbai 400614, India

**Keywords:** breast cancer, BRCA-associated, LMICs

## Abstract

Breast cancer poses a significant global health challenge, with higher incidence rates in developed countries. However, low- and middle-income countries (LMICs) suffer from higher mortality rates due to various factors, including limited screening programs, delayed diagnosis and inadequate access to healthcare and advanced treatments. Approximately 5%–10% of breast cancer cases stem from germline mutations in *BRCA*-1/2 genes. A positive *BRCA*1/2 status obtained through genetic testing significantly influences surgical and medical treatment decisions. Therefore, genetic counseling, proper surveillance and customized interventions for *BRCA*1/2 carriers are essential to maximizing the benefits of monitoring, chemoprevention and risk-reducing surgeries for breast and ovarian cancers. Identification of *BRCA* mutations also impacts treatment strategies, leading to the integration of chemotherapeutic agents like platinum-based chemotherapy and PARP inhibitors. However, implementing these advanced treatment guidelines in LMICs with complex, fragmented and underfunded healthcare systems presents numerous challenges. In this review, we explore the current status and obstacles associated with managing *BRCA*1/2-associated breast cancer in LMICs.

## Introduction 

Breast cancer poses a significant health challenge, currently ranking as the most commonly diagnosed cancer and the primary cause of cancer-related deaths among women [1]. There are substantial global variations in breast cancer incidence, largely influenced by differences in hormonal, nutritional and reproductive factors, along with socioeconomic statuses across countries [2]. The most significant rise in breast cancer incidence is observed in low- and middle-income countries (LMICs) across South America, Africa and Asia, regions traditionally known for lower incidence rates compared to industrialized nations [3–5]. This trend is frequently linked to the ‘Westernization’ of these nations, characterised by the widespread adoption of Western dietary patterns and sedentary lifestyles [6, 7]. In LMICs, hormonal risk factors such as early onset of menstruation, delayed childbirth and reduced breastfeeding are increasingly prevalent [6]. Moreover, lifestyle factors such as smoking, alcohol use and obesity are becoming more prevalent in developing nations [8]. Significantly, breast cancer tends to impact younger women in LMICs more than it does in high-income countries [9].

Women living in LMICs experience a 17% higher mortality rate compared to those in developed nations [3]. Additionally, 70% of breast cancer deaths take place in the developing world [10]. The 5-year survival rates for breast cancer in LMICs are substantially lower, ranging from 12% to 52%, compared to rates above 80% in developed nations [11, 12]. In some regions of Middle, Eastern and West Africa, the mortality-to-incidence ratio can be as high as 0.55, whereas in North America, this ratio is 0.16 [13]. The lower survival rates in LMICs are attributed to several factors, such as the absence of screening programs, delayed diagnosis, restricted access to healthcare and inadequate application of contemporary treatment methods [14].

A notable 5%–10% of newly diagnosed breast cancer cases are due to hereditary factors, mainly resulting from germline autosomal dominant mutations in the Breast Cancer Susceptibility Genes, *BRCA*1 and *BRCA*2 [15]. These genes are crucial as dynamic regulators of genomic integrity, coordinating DNA repair processes and transcriptional regulation in response to DNA damage [16]. The cumulative risk of developing breast cancer by age 70 is approximately 55%–70% for individuals carrying *BRCA*1 mutations and 45%–70% for those carrying *BRCA*2 mutations [17]. However, the exact prevalence of hereditary breast cancers remains unclear in LMICs due to limited cancer registry data [18]. Although studies from India suggest a higher occurrence of germline *BRCA*1/2 mutations among breast cancer cases, it is uncertain whether this finding can be generalised to other LMICs [19, 20]. This review aims to explore the current status and challenges associated with managing *BRCA*1/2-associated hereditary breast cancer in LMICs.

## Challenges in identifying and testing for BRCA1/2 mutations in populations at risk in LMICs

Early identification of individuals at risk for *BRCA*1/2 mutations is crucial for effective management and prevention of hereditary breast cancer. However, LMICs face numerous challenges in genetic counseling and treatment-focused genetic testing.

Many LMICs have a shortage of professional genetic counselors, national guidelines for genetic screening and insurance coverage for genetic counseling services [21]. Both patients and healthcare providers often lack awareness of the importance of genetic counseling. Despite specific guidelines for referring patients with suspected hereditary breast cancer, many eligible women are not referred for genetic counseling and screening, even in developed countries [22]. The utilisation of genetic counseling services in LMICs is notably limited, primarily due to intricate ethical, sociocultural and religious factors [23]. A study conducted in India highlighted a widespread lack of awareness regarding hereditary breast cancer and genetic counseling among breast cancer patients [24].

Access to cancer genetics testing services is constrained in LMICs mainly because of financial obstacles. Public healthcare systems in many LMICs are underfunded and disjointed, leaving patients responsible for the expenses associated with genetic counseling and testing. Insurance and public health funding agencies in LMICs typically do not cover the costs of genetic testing, and the health insurance coverage for the population is often insufficient [25]. Testing for germline *BRCA* mutation costs $200–400 in India, while the per-capita expenditure on health was nearly $60 [26, 27]. Besides privately run laboratories and hospitals driven by market forces and lacking standardised operating procedures, geographic and logistical challenges contribute to the subpar quality of genetic testing services in LMICs [23, 28]. Poor genetic literacy among healthcare personnel and the community exacerbates these challenges, leading to the underutilisation of genetic services [29, 30]. In LMICs, where social stigma and cultural conflicts are prevalent, a considerable number of women are uneducated and financially reliant on their families. A diagnosis of hereditary cancer can impose significant psychological burdens and negatively impact their quality of life [31, 32].

There is a lack of universally accepted guidelines for genetic counseling, testing and managing *BRCA* mutation carriers in LMICs. Genetic testing has become increasingly intricate, with commercial laboratories providing diverse testing options in various packages. These complexities present significant challenges for healthcare professionals in integrating genetic testing into clinical protocols [33].

Genetic counseling is recommended for individuals with *BRCA*1/2 mutations because of the well-documented advantages of surveillance, chemoprevention and risk-reducing surgeries in managing breast and ovarian cancer [34]. A positive *BRCA*1/2 genetic test outcome significantly influences the decisions regarding surgical and medical interventions [35]. The primary surgical decision involves choosing between breast-conserving surgery and bilateral risk-reducing mastectomy (RRM). Furthermore, *BRCA* carriers should consider risk-reducing bilateral salpingo-oophorectomy (RRBSO) to decrease ovarian cancer risk significantly [35, 36].

## Challenges in screening BRCA-associated breast cancer in LMICs

Early detection and timely access to effective cancer therapy are critical measures in reducing breast cancer-related mortality. The prognosis of breast cancer improves when it is detected early, while the disease remains localised and has not spread to other parts of the body [37]. For women aged 40 years and older with an average risk of developing breast cancer, screening mammography continues to be the recommended approach [38]. Studies have demonstrated that it can decrease breast cancer mortality by 20%–35% among women aged 50–69 years and slightly less among those aged 40–49 years [39].

*BRCA*-related breast cancers are distinguished by their early onset. Research indicates that the median age of breast cancer onset is 42 years for *BRCA*1 carriers and 48 years for *BRCA*2 carriers [40]. Due to the higher breast tissue density typically observed in younger women, mammography may be less effective as a screening strategy for detecting *BRCA*1/2-associated cancers, which often occur at younger ages [41]. For *BRCA*1/2 carriers, magnetic resonance imaging (MRI) of the breasts emerges as a more sensitive albeit less specific screening tool than mammography [42]. In light of this, the American Cancer Society recommends a combination of annual mammography and breast MRI for breast cancer surveillance in *BRCA* mutation carriers [43]. This combined approach not only improves the sensitivity and specificity of breast cancer detection but also facilitates earlier detection of breast cancer in *BRCA*1/2 mutation carriers [44]. Before surgical planning, it is crucial to assess both the ipsilateral and unaffected contralateral breasts through imaging, as *BRCA*1/2 patients are at higher risk of multifocal or multicentric breast cancer [45].

In LMICs, the implementation of mammography screening is hindered by significant financial and technical challenges. It requires high-quality machines, well-trained radiologists and skilled technicians [46]. Studies demonstrate a marked disparity in the availability of mammography equipment across different income brackets: less than 16% in low-income countries, 23% in LMICs, 48% in upper-middle-income countries and 78% in high-income countries [47]. Moreover, the scarcity of MRI machines compounds the issue, exacerbated by a severe shortage of medical physicists, radiographers and radiologists. Low-income countries, for instance, have only 1.9 radiologists per million people, whereas high-income countries have 97.9 radiologists per million [48]. In facilities lacking mammography and MRI equipment, clinicians may resort to clinical breast examinations or ultrasound scans despite their suboptimal effectiveness as screening modalities [49].

Beyond the availability of screening facilities, ensuring high participation rates among eligible women is equally crucial. The World Health Organisation recommends a minimum 70% participation rate in screening programs to reduce mortality [50]. However, low compliance with screening tests poses a significant hurdle in LMICs. For instance, a screening trial involving clinical breast examination in the Philippines was terminated due to women's reluctance to participate in follow-up despite extensive counseling, transportation and home visitation efforts [51]. In another screening study in Egypt, over half of the women recalled for further evaluation were lost to follow-up [52]. Reasons for non-compliance in LMICs range from avoidance and denial to fatalism and financial constraints [53]. While early diagnosis through screening offers a meaningful survival benefit, the economic burden of biopsies and follow-up examinations could strain already fragmented healthcare systems in many LMICs [54].

Additionally, a lack of awareness among healthcare providers regarding which patients should undergo evaluation poses another barrier to effectively managing individuals with hereditary cancers. For example, *BRCA*-related cancers often present at a younger age and may be misdiagnosed by primary care physicians, who may not recommend a triple test, including biopsy [55]. Socioeconomic factors also contribute to delayed breast cancer diagnoses in LMICs, where women may lack awareness of cancer symptoms or encounter obstacles accessing timely investigations due to financial constraints, geographical remoteness from healthcare facilities and extended wait times in public hospitals [56]. Cultural and religious beliefs further complicate breast cancer control and prevention efforts, with fatalistic attitudes prevalent among women in LMICs, perceiving breast cancer as predestined or divine retribution [57]. In communities like Bangladesh, breast cancer may be viewed as a curse or punishment for sins [58], while in Pakistan, diagnosis may lead to social ostracism, psychological stress and family discord [59]. Stigmatisation also deters women from seeking medical help promptly, particularly for *BRCA*1/2-associated breast cancers affecting younger, financially dependent women in LMICs, exacerbating delays in diagnosis and impeding treatment outcomes [60].

## Challenges in diagnosis and treatment of BRCA-associated cancers in LMICs

In many LMICs, the absence of comprehensive population-based cancer registries hinders critical information regarding anatomic stage, receptor status, prognostic markers and genetic determinants [61]. Obtaining a prompt and thorough histopathological review poses a significant challenge in LMICs due to limited access to high-quality tissue processing facilities, prognostic marker evaluation and adequately trained pathology personnel [62]. At the time of breast cancer diagnosis, conducting a core-needle biopsy is recommended as it provides sufficient tissue for assessing invasive versus *in-situ* status and performing immunohistochemistry (IHC) for estrogen and progesterone hormone receptors (ER/PR) and human epidermal growth factor 2 (HER2/neu) testing [63]. It is noteworthy that triple-negative breast cancer, characterised by negative ER/PR and HER2/neu status, accounts for a significant proportion of *BRCA*1-positive and *BRCA*2-positive patients [64]. *BRCA*1-positive patients typically exhibit a high nuclear grade under microscopy, underscoring the importance of accurately determining the ER/PR/HER2 status and nuclear grade through access to high-quality histology and IHC facilities [65].

Ensuring adequate quality control in handling biopsy or excision specimens remains a considerable challenge in LMICs, where the pathology team often struggles with limited control over cold ischemia time, ideally less than 60 minutes, to prevent degradation of critical biomarker proteins and false-negative results in IHC [66, 67]. Additionally, longer turnaround times (TATs) for pathology results, ranging from weeks to months, as opposed to the recommended two-business-day TAT for biopsy specimens by the College of American Pathologists, have been reported in LMICs, potentially leading to inappropriate therapy choices or disease progression resulting in stage migration [68–70]. Timelier pathology reports are essential for intra-operative frozen section samples to identify patients who may benefit from more radical surgery, emphasizing the importance of an adequate pathology workforce in LMICs where availability varies widely across regions [71, 72].

With limited resources for early detection, most breast cancer patients in LMICs present with advanced-stage disease (stages III and IV), a considerably higher proportion compared to developed countries [8, 73, 74]. *BRCA*-associated cancers, known for their aggressive biology and significant tumour burden, often require a standard treatment protocol of neoadjuvant chemotherapy followed by surgery and adjuvant radiotherapy or targeted therapy tailored to individual tumour characteristics [75, 76]. Despite the potential benefits of breast-conserving surgeries, their utilisation remains low in LMICs due to advanced disease presentation and deficiencies in surgical expertise and radiotherapy facilities [77, 78].

Chemotherapy regimens for managing *BRCA*-mutated cancers are advancing quickly, with platinum agents and adjuvant capecitabine demonstrating potential for improving survival rates [79–81]. However, these options pose challenges in LMICs due to nutritional deficiencies and higher toxicity risks [82]. Therefore, in LMICs, careful patient selection is necessary for these newer treatment approaches. Additionally, advanced therapies such as PARP inhibitors and immunotherapy remain largely inaccessible due to their high costs, further widening the treatment gap in these regions [82, 83].

Addressing fertility and pregnancy-related concerns is especially important for *BRCA*-associated breast cancer patients, as many are within the reproductive age group. Research conducted among healthcare professionals and patients in LMICs has shown a lack of understanding, practices and attitudes regarding fertility preservation and post-treatment pregnancy in young women diagnosed with breast cancer [84, 85]. Research conducted among young women diagnosed with breast cancer in India revealed that only 8% of patients underwent fertility preservation, while 13% experienced significant postmenopausal symptoms following treatment [86]. Unfortunately, these vital concerns are frequently neglected in LMICs.

## Challenges in risk-reducing strategies of BRCA-associated cancers in LMICs

Recommendations for lowering breast cancer risk in *BRCA*1/2 mutation carriers include regular surveillance, risk-reducing surgeries and chemoprevention [87]. These strategies should be personalised based on patients' preferences, quality of life concerns and life expectancy. While prophylactic RRM markedly decreases cancer risk, it does not eliminate the possibility of breast cancer. Multiple studies, both retrospective and prospective, suggest that bilateral mastectomy reduces the risk of breast cancer by approximately 90% in *BRCA*1/2 carriers [88]. RRBSO is an effective strategy for reducing the risk of ovarian and fallopian tube cancer in *BRCA*1/2 carriers [89]. However, its impact on reducing breast cancer risk in *BRCA*1 carriers remains a topic of debate [90, 91]. Furthermore, the uptake of RRM and RRBSO remains low in LMICs due to the absence of reconstructive surgery services in the public sector [92]. A study conducted in Pakistan found that many *BRCA* carriers declined RRM due to financial constraints, misunderstandings about their health status, familial or spousal pressures, worries about body image and societal perceptions and concerns about potential complications and their impact on quality of life [93].

Hormonal therapy for risk reduction, such as tamoxifen, is generally considered less effective compared to surgical interventions. Tamoxifen has shown effectiveness in *BRCA*2 carriers, who often have estrogen receptor-positive tumours, unlike *BRCA*1 tumours, which typically present as triple-negative [94]. Moreover, most studies examining preventive strategies originate from developed countries, with limited research available from LMICs. This scarcity of evidence undermines the confidence of healthcare providers in LMICs when it comes to making referrals for genetic counseling, testing and risk-reduction procedures [33].

## Proposed strategies for managing BRCA-associated breast cancer in LMICs

The distinct challenges faced by LMICs demand a customized approach. Table 1 delineates these obstacles and proposes potential solutions. Initiatives such as implementing universal health coverage, developing national cancer care plans to guarantee access to essential diagnostic and treatment resources and establishing comprehensive population-based cancer registries are critical for strengthening healthcare systems in LMICs. These registries will provide valuable data on disease burden and facilitate targeted interventions tailored to specific populations, including individuals with *BRCA*1/2 germline mutations.

Tailored breast cancer awareness initiatives that are culturally sensitive and focus on identifying risk factors and promoting early cancer detection are essential, with active involvement from advocacy groups. Effective implementation of breast cancer programs relies on a comprehensive assessment of local contexts, including disease prevalence, existing infrastructure, resource availability and sociocultural factors influencing women's participation [63]. Disseminating breast cancer awareness campaigns using culturally appropriate materials in local languages can help mitigate social stigma and cultural barriers related to breast cancer diagnosis and treatment. Embracing technology-driven solutions such as telemedicine, telepathology and affordable point-of-care molecular diagnostics can improve patient care in LMICs with complex and fragmented healthcare systems [95]. Mobile imaging technologies and screening camps offer cost-effective and accessible avenues for early cancer detection with quicker TAT [37]. Strengthening screening services involves ensuring adequate imaging equipment, trained personnel and pathology services. In regions with limited high-cost screening modalities, biennial clinical breast examinations conducted by trained primary health workers can be a feasible screening strategy [96].

Promoting access to essential cancer treatments such as surgery, radiotherapy and chemotherapy at subsidized rates in public hospitals is highly recommended. Virtual multidisciplinary tumour boards can significantly improve treatment decisions and patient outcomes, especially in regions without specialized cancer centers [97]. Educating patients about clinical trial opportunities and addressing barriers to participation can promote clinical research and expand access to innovative treatment options.

International collaboration and cross-border medical research utilising open-access, de-identified genomic databases offer promise in advancing care for genetically predisposed breast cancer patients in LMICs [98]. Raising awareness about genetic counseling among healthcare providers and expanding the number of genetic counselors trained in risk reduction strategies are essential steps. Quality control measures and standard operating procedures in laboratories are crucial for ensuring the ethical and practical use of genetic testing with reliable reporting. LMICs must develop comprehensive guidelines for genetic testing, risk reduction strategies and follow-up protocols tailored to the local context. Advocacy efforts are vital to achieving equitable access to genetic testing and care, mobilizing support from caregivers, patients and families to address barriers posed by government facilities and insurance providers, including cost concerns, limited awareness and competing priorities.

In conclusion, addressing the multifaceted challenges of managing *BRCA*-associated breast cancer in LMICs requires a multifaceted approach encompassing tailored awareness initiatives, strengthened healthcare infrastructure, access to affordable treatments and robust international collaboration. By implementing these strategies, LMICs can significantly improve breast cancer outcomes and ensure equitable care for all patients, including those with *BRCA*1/2 germline mutations.

## Conflicts of interest/disclosure

There are no relevant conflicts of interest to declare.

## Funding

No external sources of funding.

## Figures and Tables

**Table 1.** Critical challenges and suggested solutions.

Challenge	Possible solutions
Lack of population-specific information on hereditary cancers	Development of hospital-based cancer registries
Poor coverage of breast cancer screening	Mobile screening camps, media awareness campaigns, investment in high-quality machines and training of personnel
Shortage of genetic counsellors	Tele-genetics, training of genetic counsellors, improving awareness of genetic counselling among healthcare workers
Sociocultural and societal barriers to genetic testing	Mass education and advocacy campaign
Financial constraints for genetic testing	Coverage by insurance companies, government schemes
Laboratory – variability in technologies for genetic testing	Development of quality control processes and standard operating procedures
Indiscriminate genetic testing without appropriate genetic counselling	Education of healthcare professionals and adoption of guidelines for testing
Lack of access to cancer diagnosis and treatment	Universal health coverage, national cancer care plans, subsidized treatment schemes, virtual multidisciplinary tumour boards
Low availability of newer drugs like PARP inhibitors	Subsidized pricing, more insurance coverage
Intensification of chemotherapy with specific agents like platinum agents, capecitabine	Careful patient selection, further research to look at efficacy in LMIC population
Menopausal symptoms and fertility issues	Counselling regarding potential complications of cancer therapy and fertility preservation techniques
